# The Senghenydd Disaster

**Published:** 1913-11-29

**Authors:** 


### THE SENGHENYDD DISASTER.

Colonel Bruce Vaughan reported at the monthly meeting of the board of management of King Edward VII.'s Hospital, Cardiff, that a Cardiff gentleman, Mr. G. A. Lundie, had inquired of him what it had cost the hospital to treat the patients who were sent down as a result of the recent explosion at Senghenydd. The secretary and the resident medical officer calculated £18 14s., whereupon Mr. Lundie had forwarded a cheque for this amount, with an appreciative letter. In connection with the disaster, the secretary reported that fifty-seven beds were immediately prepared for the reception of the injured, and Mr. Lynn Thomas had also offered to place six beds in Bedford House Private Hospital at their disposal. The appalling nature of the calamity was evidenced by the sad fact that only six beds were eventually required. Colonel Bruce Vaughan further announced that one of the oldest and most respected of the members of the board—Mrs. Peter Price—had sent a cheque for 100 guineas to mark the occasion of her birthday. He said that Mrs. Peter Price had proved a most loyal and helpful friend of the hospital for a longer period than probably any other living person.

